# Prescribed Versus Preferred Intensity Resistance Exercise in Fibromyalgia Pain

**DOI:** 10.3389/fphys.2018.01097

**Published:** 2018-08-10

**Authors:** Roberta P. da Cunha Ribeiro, Tathiane C. Franco, Ana J. Pinto, Marco A. G. Pontes Filho, Diogo S. Domiciano, Ana L. de Sá Pinto, Fernanda R. Lima, Hamilton Roschel, Bruno Gualano

**Affiliations:** ^1^Rheumatology Division, Hospital das Clinicas HCFMUSP, Faculdade de Medicina, University of São Paulo, São Paulo, Brazil; ^2^Applied Physiology and Nutrition Group, Faculdade de Medicina, University of São Paulo, São Paulo, Brazil

**Keywords:** chronic pain, hyperalgesia, non-pharmacological intervention, physical activity, strength

## Abstract

Exercise is the treatment of choice for fibromyalgia (FM), but little is known about resistance exercise prescription to modulate pain in this condition. This study aimed to compare the effects of different resistance exercise models, comprising self-selected or prescribed intensity, on pain in FM patients. In a cross-over fashion, 32 patients underwent the following sessions: (i) standard prescription (STD; 3 × 10 repetitions at 60% of maximal strength); (ii) self-selected load with fixed number of repetitions (SS); (iii) self-selected load with volume load (i.e., load × sets × repetitions) matched for STD (SS-VM); and (iv) self-selected load with a free number of repetitions until achieving score 7 of rating perceived exertion (SS-RPE). Pain, assessed by Visual Analogic Scale (VAS) and Short-Form McGill Pain Questionnaire (SF-MPQ), was evaluated before and 0, 24, 48, 72, and 96 h after the sessions. Load was significantly lower in SS, SS-VM, SS-RPE than in STD, whereas rating perceived exertion and volume load were comparable between sessions. VAS scores increased immediately after all sessions (*p* < 0.0001), and reduced after 48, 72, 96 h (*p* < 0.0001), remaining elevated compared to pre-values. SF-MPQ scores increased immediately after all exercise sessions (*p* = 0.025), then gradually reduced across time, reaching baseline levels at 24 h. No significant differences between sessions were observed. Both prescribed and preferred intensity resistance exercises failed in reducing pain in FM patients. The recommendation that FM patients should exercise at preferred intensities to avoid exacerbated pain, which appears to be valid for aerobic exercise, does not apply to resistance exercise.

## Introduction

Fibromyalgia (FM) is a chronic syndrome of unknown etiology characterized by widespread pain, physical dysfunction, fatigue, psychological distress resulting in pain-related catastrophizing, cognitive dysfunction, sleep, and mood disturbances (Fietta et al., 2007; Hauser et al., 2008). Drug therapy (e.g., antidepressants, opioids, sedatives, and antiepileptic medications) has modest efficacy and may often cause adverse effects (Nuesch et al., 2013), whereas non-pharmacological interventions, in particular exercise training, appear to promote consistent therapeutic benefits in this syndrome (Goldenberg et al., 2004; Busch et al., 2007). In fact, the European League Against Rheumatism (EULAR) revised recommendations for the management of FM indicated that, based on meta-analyses, the only “strong for" therapy-based recommendation was exercise (Macfarlane et al., 2017).

Aerobic exercise has been largely recommended to improve well-being, physical capacity and functionality during management of FM (Newcomb et al., 2011). Studies corroborating these beneficial effects are numerous, and evidence-based guidelines for the prescription of aerobic exercise programs (e.g., intensity, frequency, duration, length) have been reported (Hauser et al., 2010). In contrast, the literature involving resistance training is still limited. Yet, a systematic review concluded that there is evidence (although rated as low quality) suggesting that moderate- to high-intensity resistance training can improve multidimensional function, muscle strength, tenderness, and pain in FM patients (Busch et al., 2013). In contrast to aerobic exercise, however, little is known about the effective prescription of resistance exercise to improve FM symptoms, in particular pain (Nelson, 2015).

Perhaps the major challenge with regard to the management of FM through exercise is the low adherence rates of patients to exercise training programs (Meyer and Lemley, 2000; van Santen et al., 2002; Schachter et al., 2003; Busch et al., 2008). There is evidence that pain has been associated with poor exercise tolerance in FM patients (Culos-Reed and Brawley, 2000), thus constituting an important barrier for them to engage in physical activity (de Gier et al., 2003). Interestingly, studies suggest that pain modulation by exercise may be different between FM patients and healthy controls. For instance, while exercise is thought to increase pain threshold in healthy individuals, this effect may be absent in FM (Mengshoel et al., 1995b; Kosek et al., 1996; Staud et al., 2005; Lannersten and Kosek, 2010; Bement and Sluka, 2016). In fact, there are conflicting data suggesting that FM patients may experience either exercise-induced hypoalgesic (Kadetoff and Kosek, 2007; Staud et al., 2010) or hyperalgesic (Mengshoel et al., 1995b; Meyer and Lemley, 2000; Kadetoff and Kosek, 2007; Kayo et al., 2012) responses (i.e., decrease and increase in pain, respectively, following a noxious stimulus), which might be dictated by exercise type and/or intensity.

In this regard, it has been suggested that the use of preferred intensity may positively modulate pain in FM as self-selected intensity may allow patients to adjust the intensity of exercise as necessary to remain comfortable and to minimize possible exacerbations in pain during exercise (Newcomb et al., 2011). In a previous study involving aerobic exercise, both preferred and prescribed intensities (∼45 and ∼62% of age-adjusted maximum intensity, respectively) ensued similar analgesic effects in mild FM patients, despite the lower intensity and perceived exertion in the self-selected exercise session. This led the authors to suggest that preferred, rather than prescribed, aerobic exercise should be recommended seeking to improve exercise adherence (Newcomb et al., 2011). However, whether this notion extends to resistance exercise remains to be determined. Considering that resistance exercise may confer numerous therapeutic benefits for FM patients (Busch et al., 2013), it is important to investigate the role of distinct models of resistance training on the modulation of pain.

This study aimed to compare the acute effects of prescribed and preferred intensity resistance exercises on pain in FM patients. We hypothesized that (i) self-selected load would be lower than that of standard prescription; (ii) all exercise models would produce a beneficial effect on pain; (iii) self-selected intensity exercise models would lead to lower perceived exertion, possibly resulting in greater reduction of pain when compared with the standard prescription model.

## Materials and Methods

### Study Design and Participants

This was a prospective, crossover, randomized, counterbalanced, clinical study conducted in São Paulo, Brazil (Clinical Hospital, School of Medicine, University of São Paulo, Brazil). The sample consisted of 32 female FM patients (age between 20 and 55 years). All patients fulfilled the American College of Rheumatology (2011) criteria for FM, whose diagnostic variables include a widespread pain index (WPI), a measure of the number of painful body regions, and a symptom severity (SS) scale, composed by categorical scales for cognitive symptoms, unrefreshed sleep, fatigue, and number of somatic symptoms (Wolfe et al., 2011).

Exclusion criteria included: cardiac and pulmonary involvement, and musculoskeletal and joint disorders, which could preclude exercise testing or session; severe neuropsychological disorders; and engagement in any resistance training program in the last 6 months prior to the study.

The study was approved by Ethics Commission for Analysis of Research Projects (CAPPesq) from the Clinical Hospital (School of Medicine, University of São Paulo, Brazil) and all participants signed the written informed consent form.

### Experimental Design

Patients attended our laboratory on eight occasions. During the first four visits, patients were familiarized with lower-limb (i.e., leg press) and upper-limb (i.e., bench press) 1-repetition maximum (1-RM) tests, as well as with the different exercise models. Afterward, during the visits five to eight, patients underwent the following experimental sessions in a randomized cross-over fashion: (i) standard prescription according to the American College of Sports Medicine (STD; 6 × 10 repetitions 60% of 1-RM); (ii) self-selected intensity with fixed number of repetitions (i.e., 6 × 10 repetitions) (SS); (iii) self-selected intensity with volume-load (repetitions × sets × intensity) matched for STD (SS-VM). This session was designed to dissociate the effect of intensity and volume load on pain; and (iv) self-selected load with a free number of repetitions until achieving score 7 (i.e., very hard) in the rating of perceived exertion (SS-RPE).

**FIGURE 1:**
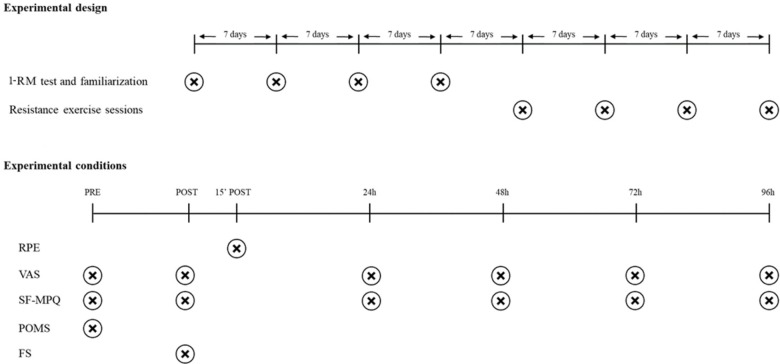
Study design. ^∗^Seven day-interval between each experimental condition. Abbreviations: 1-RM, 1-repetition maximum; RPE, Rating of Perceived Exertion; VAS, Visual Analogic Scale; SF-MPQ, Short-Form McGill Pain Questionnaire; POMS, Profile of Mood States; FS, Feeling Scale.

In order to avoid any circadian effect on pain, individual exercise sessions occurred at the same time of the day. In addition, carryover effects were prevented by giving the patients a 7-day interval between each experimental condition (**Figure 1**).

Before each resistance exercise session, patients completed pain and mood states questionnaires. Afterward, patients underwent the experimental session, which comprised 3 sets of leg press exercise and 3 sets of bench press exercise. Immediately after the experimental session, patients completed pain questionnaires and an affective valence scale, and 15 min later, they reported their session’s RPE. Pain was also assessed at 24, 48, 72, and 96 h following exercise (**Figure 1**).

### Muscle Strength

Patients performed 3 familiarization sessions, 7 days apart from each other, before the 1-RM test in leg press and bench press exercise. Prior to the 1-RM test, two light warm-up sets, interspersed by 2-min intervals, were performed. Subsequently, participants achieved 1-RM for each exercise in 1–5 attempts, interspersed by 3-mi intervals (Brown and Weir, 2001). 1-RM tests were conducted by one experienced researcher and verbal encouragement was provided during testing sessions. The coefficient of variation (CV) for 1-RM tests were <5% for all patients.

### RPE

The RPE was assessed by the Borg CR-10 RPE scale (Borg, 1998). Patients graded their perceived exertion 15 min after each resistance exercise session, where zero represents no effort (i.e., rest) and 10 represents the maximal effort (i.e., the most stressful exercise ever performed). Patients were asked to report any number on the scale to rate their overall effort. RPE was assessed by one experienced researcher and participants were familiarized to this tool.

### Pain

Pain was assessed by the same non-blinded researcher (R.P.C.R.) using the Visual Analogic Scale (VAS), through which patients should grade their pain using a 10-point scale, where zero means no pain and 10 means severe or unbearable pain, and by the Short-Form McGill Pain Questionnaire (SF-MPQ) (Melzack, 1987), which ranges from 0 to 45 (the higher the SF-MPQ values, the greater the pain levels).

### Mood States and Affective Valence

Mood states were assessed by the Profile of Mood States (POMS) (Viana et al., 2001), which ranges from 0 to 200. The higher the score, the greater the mood disturbance. Affective valance to resistance exercise sessions were assessed by the Feeling Scale (FS) (Hardy and Rejeski, 1989), which is an 11-point scale ranging from −5 (very bad) to +5 (very good).

### Sample Size and Statistical Analysis

As there was a lack of data to calculate sample size for the main outcome (i.e., pain responses to resistance training), the number of participants was chosen based on the feasibilities, such as funds, capacity of research staff and facility, and available patients, in line with current recommendations (Bacchetti et al., 2008; Bacchetti, 2010). A number multiple of four was necessary to ensure a proper randomization between the experimental conditions, to avoid any order effect.

Mixed model analysis was performed for all dependent variables. For exercise load, volume load and repetitions, RPE, POMS, and FS, experimental condition (STD, SS, SS-VM, and SS-RPE) was determined as fixed factor and patients as a random factor. For VAS and McGill, experimental condition (STD, SS, SS-VM, and SS-RPE) and time (pre-exercise, post-exercise, 24, 48, 72, 96 h) was determined as fixed factors and patients as a random factor. In case of significant *F*-values, a *post hoc* test with Tukey’s adjustment was performed. Analyses were performed using the SPSS, v. 17.0, and the SAS, v. 9.3, for Windows. The level of significance was set at *p* ≤ 0.05. Data are presented as mean ± standard deviation.

## Results

**Table 1** depicts demographic data, disease parameters, current clinical treatment, comorbidities, muscle strength, and pain in FM patients.

**Table 1:** Demographic data, disease parameters, current clinical treatment, comorbidities, muscle strength, and pain in FM patients.

Variable	Participants (*n* = 32)
Age (years)	47.8 ± 13.7
BMI (m/kg^2^)	26.5 ± 3.1
Time elapsed since diagnoses (years)	9.4 ± 6.6
**Drugs: *n* (%)**	
No drugs	03/32 (9.3%)
Antidepressants	21/32 (65.6%)
Analgesic	11/32 (34.3%)
Muscular relaxant	17/32 (53.1%)
NSAIDS	05/32 (15.6%)
Opioids	04/32 (12.5%)
**Comorbidities: *n* (%)**	
Osteoarthritis	02/32 (6.2%)
Arterial hypertension	09/32 (28.1%)
Dyslipidemia	04/32 (12.5%)
Diabetes mellitus	05/32 (16.6%)
Osteoporosis	06/32 (18.7%)
Asthma	01/32 (3.1%)
1-RM Leg press (kg)	144.5 ± 47.6
1-RM Bench press (kg)	27.2 ± 6.3
SF-MPQ pre	22.6 ± 15.8
VAS pre	4.2 ± 2.7


*Data expressed as mean ± SD or n (percentage of the sample). Abbreviations: (1-RM) 1-repetition maximum, (RPE) Rating of Perceived Exertion, (VAS) Visual Analogic Scale, (SF-MPQ) Short-Form McGill Pain Questionnaire.*

**FIGURE 2:**
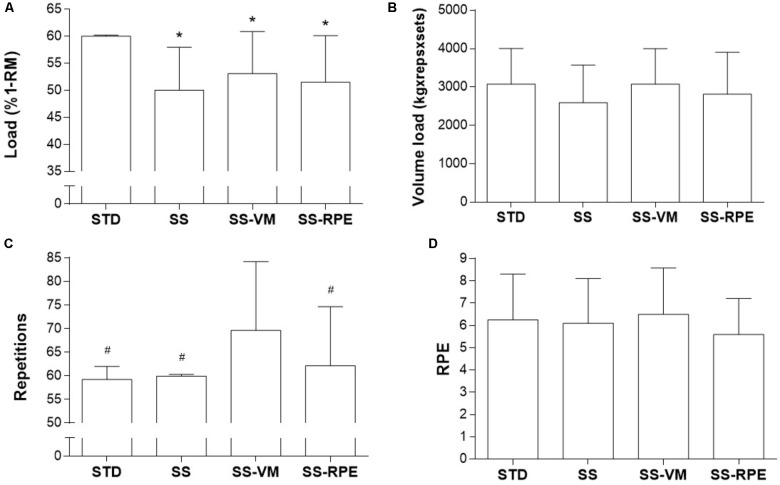
Load **(A)**, volume load **(B)**, repetitions **(C)**, and RPE **(D)** in STD, SS, SS-VM, SS-RPE sessions. Data expressed as mean and standard deviation. ^∗^*p* < 0.05 vs. STD; ^#^*p* < 0.05 in vs. SS-VM.

No order effect was noted for any dependent variable. **Figure 2** shows the data regarding load (Panel A), volume load (Panel B), repetitions (Panel C), and RPE (Panel D) for all exercise sessions. Load was significantly lower in SS, SS-VM, SS-RPE than in STD. Total repetitions were significantly higher in SS-VM than all the other sessions. Volume load and RPE were comparable between sessions.

**FIGURE 3:**
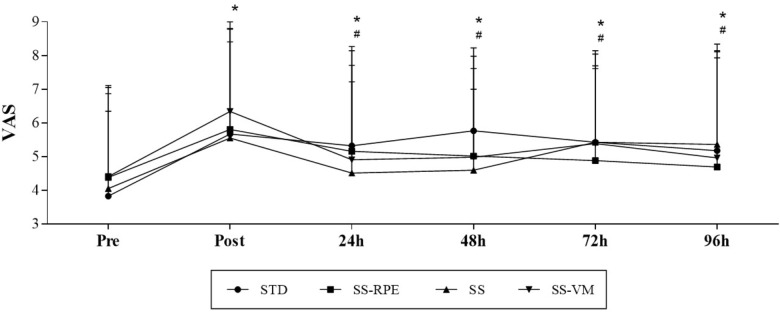
VAS scores across time in STD, SS, SS-VM, SS-RPE sessions. Data expressed as mean and standard deviation. ^∗^*p* < 0.05 vs. VAS Pre, and ^#^*p* < 0.05 vs. VAS Post.

**Figure 3** illustrates VAS data across time. VAS scores equally increased immediately after all exercise sessions (main time effect; *p* < 0.0001). Thereafter, VAS scores significantly reduced after 48, 72, 96 h (main time effect; *p* < 0.0001), but remained elevated as compared to pre-values. No significant interaction (time × session) effect was observed.

**FIGURE 4:**
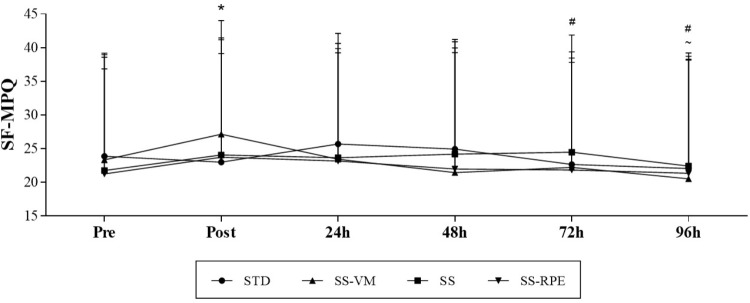
SF-MPQ scores across time in STD, SS, SS-VM, SS-RPE sessions. Data expressed as mean and standard deviation.^∗^*p* < 0.05 vs. SF-MPQ Pre, ^#^*p* < 0.05 vs. SF-MPQ Post, and ∼*p* < 0.05 vs. SF-MPQ 24.

**Figure 4** shows SF-MPQ data across time. SF-MPQ values significantly increased immediately after all resistance exercise sessions (main time effect; *p* = 0.025). SF-MPQ scores then gradually reduced across time, reaching baseline levels at 24 h. SF-MPQ scores at 72 and 96 h were significantly lower than immediately after exercise (main time effect; *p* = 0.050 and *p* = 0.002, respectively), whereas scores at 96 h were significantly lower than at 24 h (main time effect; *p* = 0.015). There was no significant interaction (time × session) effect.

POMS (STD: 144.78 ± 35.41; SS: 139.31 ± 31.36; SS-VM: 142.56 ± 29.98 and SS-RPE: 144.59 ± 35.64) and FE scores (STD: 2.80 ± 2.50; SS: 2.75 ± 2.49; SS-VM: 2.53 ± 2.52 and SS-RPE: 3.17 ± 2.12) were not significantly different between exercise sessions.

## Discussion

The aim of this study was to examine for the first time the effect of different loading schemes of resistance exercise, consisting of prescribed vs. preferred intensities, on pain in FM patients. The main findings of this study were threefold: (i) the patients preferred lower load than what was prescribed; (ii) all the resistance exercise models led to increased pain following exercise, which tended to decrease over time; (iii) the preferred intensity exercises were as ineffective as the prescribed intensity exercise to reduce pain. These findings can inform new evidence-based recommendations and guide future research on exercise prescription in FM.

It has been shown that FM patients usually experience more exercise-induced pain and exertion than healthy controls (Mengshoel et al., 1990; Mengshoel et al., 1995b), which cannot be explained by differences in physical fitness, exercise metabolic cost, metabolites accumulation (e.g., lactate, potassium or sodium) (Mengshoel et al., 1995a) or micro-traumas induced by exercise (Norregaard et al., 1994). While the mechanisms underlying this abnormal pain response remain elusive, the search for exercise models capable of preventing pain increase, or perhaps even reducing pain, are of upmost importance in the clinical setting (Hauser et al., 2010; Lannersten and Kosek, 2010; Naugle et al., 2012; Nijs et al., 2012; Nelson, 2015; Ellingson et al., 2016).

It has been speculated that preferred exertion models in which FM patients choose their own exercise intensity could lead to greater adherence rates (Dishman et al., 1994). This assumption is based on the well-known negative association between exercise intensity and adherence to exercise (Dishman et al., 1994). Hypothetically, the incorporation of preferred intensity could also allow patients to select loads compatible with their perception of pain, mitigating potential exacerbation in pain during the exercise session (Newcomb et al., 2011). This assumption was tested in a cross-over study in which FM patients performed 20 min of aerobic exercise (i.e., cycling) either at a self-selected intensity or at a prescribed intensity. FM patients self-selected lower exercise intensity than what was prescribed. Pain reduction over a 96 h-period was similar between the conditions, leading the authors to suggest that aerobic exercise prescription for FM patients should consider the preferred-intensity exercise model as a strategy to manage pain. However, the nature of exercise assessed in this study (i.e., aerobic) does not allow any firm extrapolation to other exercise models, in particular resistance exercise.

To address this gap, we examined the effects of different loading schemes of resistance exercise on pain in FM. Similar to aerobic exercise (Newcomb et al., 2011), FM patients self-selected lower loads for resistance exercise than what was prescribed based on the recommendations from the ACSM (∼52% vs. 60% of 1-RM, respectively). These data support the notion that FM patients may find it more comfortable to exercise at lower intensities than what has been prescribed to general population, and this should be considered while recommending physical activities for these patients. Furthermore, our findings revealed, for the first time, that FM patients chose to perform resistance exercise at an intensity rated between “hard” and very hard” on Borg’s CR-10 scale, whereas they seem to prefer between “light and somewhat light” intensities for aerobic exercise (Newcomb et al., 2011).

In contrast to aerobic exercise (Newcomb et al., 2011), resistance exercise in this study increased pain in the FM patients, regardless of the loading schemes. Importantly, allowing the patient to self-select intensity appeared to be ineffective to counteract the pain exacerbation. In fact, there is evidence that isometric exercise even at low intensities (i.e., 15 to 30% of maximal voluntary contraction) can aggravate pain in FM patients (Staud et al., 2005; Kadetoff and Kosek, 2007). Interestingly, the response to submaximal isometric exercise on pain modulation differs substantially between FM patients and healthy controls, with hyperalgesia responses in the former and hypoalgesia in the latter (Staud et al., 2005). The mechanism underlying this divergent response is not completely understood, but it was demonstrated that cutaneous and somatic pain was increased in both local and remote body areas following sustained submaximal isometric exercise in FM patients (Staud et al., 2005). This finding led the authors to speculate that muscular contraction induced widespread pain inhibitory effects, which is seen in healthy individuals, is absent in FM patients (Staud et al., 2005). Whether these abnormal central pain mechanisms in FM result from abnormal descending inhibition or excessive activation of muscle nociceptive afferents remains unclear (Staud et al., 2005).

Furthermore, the opposite acute effect of aerobic (Newcomb et al., 2011) and resistance exercise on pain modulation is intriguing. There is no sufficient evidence to assume that intensity-matched aerobic and resistance exercises would trigger essentially different analgesic mechanisms. Hence, one may speculate that intensity, or its subjective perception, rather than exercise type may play a greater role on exercise-induced analgesia. In fact, there is growing evidence showing that more intensive exercises, irrespective of being isometric- or aerobic-oriented, may lead to hyperalgesia, whereas low-to-moderate exercises may result in hypoalgesia in patients with chronic diffused pain (Naugle et al., 2012). In these patients, vigorous exercise-induced pain is thought to be due to abnormal descending inhibition or excessive activation of muscle afferents (Vierck et al., 2001; Staud et al., 2005); in case of resistance exercise, diminished blood flow may also play a role in exercise-induced hyperalgesia (Larsson et al., 1999; Elvin et al., 2006). This notion is apparently in contrast to our results, since the resistance exercise sessions characterized by self-selected lower intensities (as compared to the prescribed intensity) did not mitigate pain. However, one may note that our patients self-selected a substantially higher intensity when compared to the aerobic-exercised patients (Newcomb et al., 2011), as rated by the perceived exertion scale. Likewise, our resistance exercise session guided by RPE was also perceived to be more intensive than that of the patients who performed aerobic exercise at a preferred intensity (Newcomb et al., 2011) (“very hard” vs. “light and somewhat hard,” respectively). Thus, one may speculate that the relatively higher perceived intensities tested in this study, despite being considered “light” for healthy individuals (Newcomb et al., 2011), may have exacerbated pain in our FM patients. Our data also suggest that self-selecting training load may not be an effective strategy to ensure an adequate resistance exercise intensity able to reduce pain (or even to improve affection), as opposing to what was shown with aerobic exercises (Hauser et al., 2010; Newcomb et al., 2011; Kayo et al., 2012; Naugle et al., 2012). Further studies should test the effect of resistance exercises at even lower intensities, possibly resulting in lower RPE, on pain modulation in FM patients.

Alternatively, the patients’ characteristics can also explain the conflicting results. Namely, our patients had higher baseline pain than those of the previous study (31) (i.e., SF-MPQ: ∼23 vs. 10, respectively), despite a similar drug regimen. In fact, it is possible to hypothesize that patients with more severe pain could experience dysfunctioning of endogenous analgesia in response to exercise (Kosek et al., 1996; Kosek and Lundberg, 2003; Lannersten and Kosek, 2010; Nijs et al., 2012). Our data support this notion and suggest that exercising painful muscles can increase generalized pain sensitivity instead of promoting analgesic effects. Resistance training has been shown to be a useful tool to improve autonomic function, strength and function, quality of life, and overall pain (Busch et al., 2013). However, dysfunctional pain regulation may limit adherence to this type of exercise, especially if may trigger pain catastrophizing, a propensity for an exaggerated pain experience that has been linked to avoidance of physical activity, deconditioning, and perpetuated pain (de Bruijn et al., 2011). The search for strategies able to counteract this response remains as a priority. In this regard, the current overall recommendation that FM patients should exercise at preferred intensities to avoid abnormal exercise-induced pain responses is not corroborated by our study, and perhaps a prescribed intensity (certainly lower than those prescribed for healthy individuals) targeting lower perceived efforts (e.g., “light” or “somewhat hard”) might be a better alternative while recommending resistance exercises. In support to this notion, *post hoc* Pearson’s correlations from the pooled data (i.e., all patients in all exercise sessions) revealed positive associations between RPE and pain immediately after exercise (VAS: *r* = 0.416, SF-MPQ: *r* = 0.450; both *p* < 0.001), suggesting that the lower the perceived effort, the lower the pain.

## Conclusion

Both prescribed- and preferred-intensity resistance exercise failed to reduce pain in FM patients. This suggests that the recommendation that FM patients should exercise at preferred intensities to avoid exacerbated pain, which appears to be valid for aerobic exercise, does not apply to resistance exercise. From a clinical standpoint, “start slowly, progress slowly” remains the current strategy of choice for resistance exercise prescription in FM, although empirical evidence is required to validate this approach.

## Author Contributions

BG and HR conceived and designed the study, performed the statistical analysis, and wrote the manuscript. RdCR, TF, AP, MPF, DD, AdSP, and FL acquired the data, and reviewed and revised the manuscript. BG, HR, and AP analyzed and interpreted the data.

## Conflict of Interest Statement

The authors declare that the research was conducted in the absence of any commercial or financial relationships that could be construed as a potential conflict of interest.
